# Enhancing Lithium Stripping Efficiency in Anode-Free Solid-State Batteries through Self-Regulated Internal Pressure

**DOI:** 10.1021/acs.nanolett.3c02713

**Published:** 2023-10-11

**Authors:** Daxian Cao, Tongtai Ji, Zhengxuan Wei, Wentao Liang, Ruobing Bai, Kenneth S. Burch, Michael Geiwitz, Hongli Zhu

**Affiliations:** † Department of Mechanical and Industrial Engineering, Northeastern University, 360 Huntington Avenue, Boston, Massachusetts 02115, United States; ‡ Department of Physics, Boston College, Chestnut Hill, Massachusetts 02467, United States

**Keywords:** anode-free, all-solid-state Lithium metal batteries, pressure regulation, stripping efficiency, interface contact

## Abstract

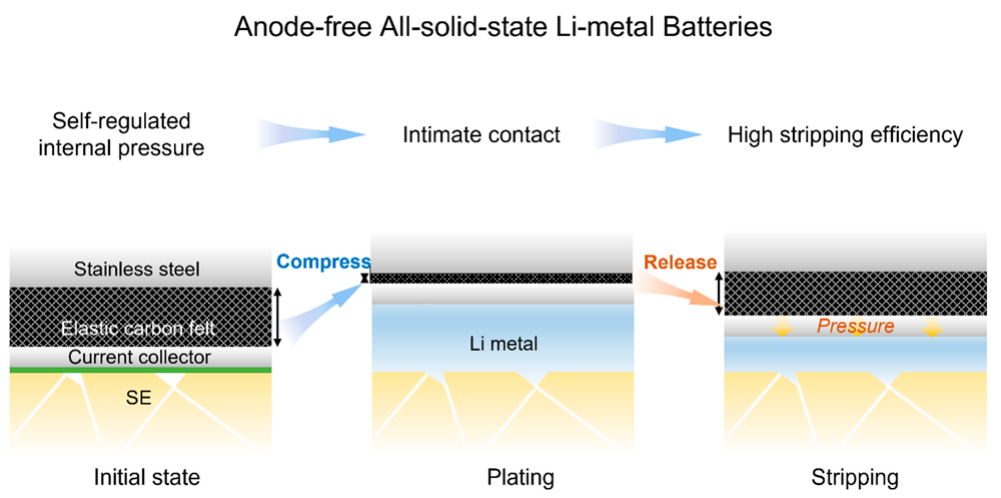

Anode-free all-solid-state lithium metal batteries (ASLMBs) promise high energy density and safety but suffer from a low initial Coulombic efficiency and rapid capacity decay, especially at high cathode loadings. Using operando techniques, we concluded these issues were related to interfacial contact loss during lithium stripping. To address this, we introduce a conductive carbon felt elastic layer that self-adjusts the pressure at the anode side, ensuring consistent lithium–solid electrolyte contact. This layer simultaneously provides electronic conduction and releases the plating pressure. Consequently, the first Coulombic efficiency dramatically increases from 58.4% to 83.7% along with a >10-fold improvement in cycling stability. Overall, this study reveals an approach for enhancing anode-free ASLMB performance and longevity by mitigating lithium stripping inefficiency through self-adjusting interfacial pressure enabled by a conductive elastic interlayer.

With the popularization of portable electronics and electric vehicles, the development of advanced energy storage techniques that are highly safe and have a high energy density would be significant.^1,2^ All-solid-state lithium metal batteries (ASLMBs), pairing solid-state electrolytes (SEs) and a lithium (Li) metal anode, are regarded as one of the most promising candidates to fulfill the requirements.^3^ The utilization of nonflammable SEs reduces the risk of thermal runaway associated with the use of liquid electrolytes. Simultaneously, the employment of a Li metal anode can overcome the limitations on the theoretical energy density of conventional Li-ion batteries.^4^ Notably, with no excess Li metal in the initial form, the anode-free configuration in which all Li comes from the Li-containing cathode can maximize the energy density of ASLMBs.^5^ However, anode-free ASLMBs are generally criticized for their low Coulombic efficiency (CE) and poor cycling life, which are much more severe than those of commonly investigated ASLMBs containing excess Li metal.^6^ Therefore, investigating the root causes of these failures and developing corresponding remedial strategies become crucial.

The unsuccessful performance of anode-free ASLMBs can be attributed to a multitude of interconnected factors. A significant obstacle in anode-free ASLMBs is the inadequate physical linkage between the SE and the anode, which serves as the initial current collector. This issue complements others inherent in Li-excess ASLMBs, including the substantial mechanical stress resulting from Li plating,^7^ the high reactivity of Li when it encounters the SEs,^8^ irregular Li-ion flux at the the anode–SE interface,^9^ the direct deposition of Li inside the SEs,^10^ etc.^11^ Unlike the better connection in Li-excess ASLMBs where the malleable Li metal can be creeping and pressed onto the SE, the metallic current collector in anode-free ASLMBs lacks a close-knit contact with the rigid SEs prior to the initial Li metal plating. This deficiency results in localized Li plating, which can readily instigate dendrite growth, subsequently causing a short circuit.^12^ Even worse, as the stripping process proceeds, the Li situated next to the SE is primarily stripped away, leading to the creation of voids between the SE and Li that adheres to the current collector. This contact loss not only causes low Li stripping efficiency with dead lithium formation but also aggravates the localization of the plating in the following cycles, resulting in a low CE and short cycling stability. Constructing and maintaining an intimate contact at the anode side are critical for developing stable anode-free ASLMBs.

Applying an external pressure, generally several or even hundreds of megapascals, is widely accepted in sulfide SE-based all-solid-state Li-ion batteries for maintaining the intimate contact between the SE and the electrodes.^13^ However, in ASLMBs, the requirement for this pressure depends on the plating/stripping processes.^14^ It has been reported that a high external pressure of 75 MPa can directly drive the Li metal to penetrate the sulfide SEs.^15^ As the Li metal plating is processed, the plating-induced stress, reported to be as high as gigapascals,^16^ coupled with the external pressure of 25 MPa, can also trigger the propagation of Li within the pores and cracks inside the SE causing the short circuit. Thus, for ASLMBs, a moderate pressure is desired for the plating process. In contrast, during stripping, the results of both experiments and simulations have proven that a high pressure enabled the creeping of Li metal benefiting the stripping of Li throughout without the formation of voids.^17^ To avert contact loss, an adjustable-pressure system is critical. This implies that for optimal regulation the pressure should be reduced during the plating stage and increased during the stripping stage. Currently, a preset initial compression typically generates the necessary external pressure. There have not been any works of rational internal pressure control implemented that align with the phases of plating and stripping reported so far.

To establish an internal pressure adjustment, herein, for the first time, we proposed enhancing the Li metal stripping efficiency in anode-free ASLMBs through self-regulation of the pressure at the anode side enabled by an elastic layer, and we demonstrated this mechanism with a compressible carbon felt. The anode-free ASLMBs were fabricated on the basis of a single-crystal high-nickel cathode, LiNi_0.8_Mn_0.1_Co_0.1_O_2_, and a sulfide SE, Li_5.4_PS_4.4_Cl_1.6_, exhibiting a high ionic conductivity of 7.8 mS cm^–1^.^18^ A piece of compressible carbon felt was placed between the current collector and a stainless steel (SS) rod used for applying external pressure. In anode-free ASLMBs, we investigated the effects of the carbon felt on the Li metal stripping efficiency, interface contact, and interface impedance evolutions. In combination with electrochemical measurement and pressure monitoring, we analyzed the critical current densities (CCDs) related to the plating and stripping processes. The mechanical properties of the carbon felt were also evaluated for a better understanding of the battery behaviors. As a result, with the addition of the carbon felt, the anode-free ASLMB delivered a significantly enhanced initial Coulombic efficiency (ICE) from 58.3% to 83.7% and a high CCD of 1.0 mA cm^–2^.

The timely adjustment of the pressure is crucial for anode-free ASLMBs. Figure 1 shows the anode-free ASLMBs with and without self-regulated pressure during various stages of plating and stripping. In traditional anode-free ASLMBs (Figure 1a), the current collector is directly compressed onto the SE by a pressing pillar used to apply a fixed initial compression to the ASLMBs. During the plating process, the Li metal experiences a significant volume expansion, which increases the internal pressure. The plated Li metal occupies the voids present on the SE surface. During the stripping process, the Li metal attached to the SE is primarily stripped because the plating/stripping predominantly occurs at the interface where both ion accessibility and electron accessibility are best. Consequently, voids were generated in the sites previously occupied by the plated Li metal. Due to the absence of sufficient pressure during the stripping process, the Li metal that remains unstripped at the anode side forms a porous structure, leading to low Coulombic efficiency and a significant increase in interface impedance. In the subsequent plating process, the inadequate contact between the SE and Li metal results in a localized current and ion flux concentration, which readily triggers the growth of dendrites.

**Figure 1:**
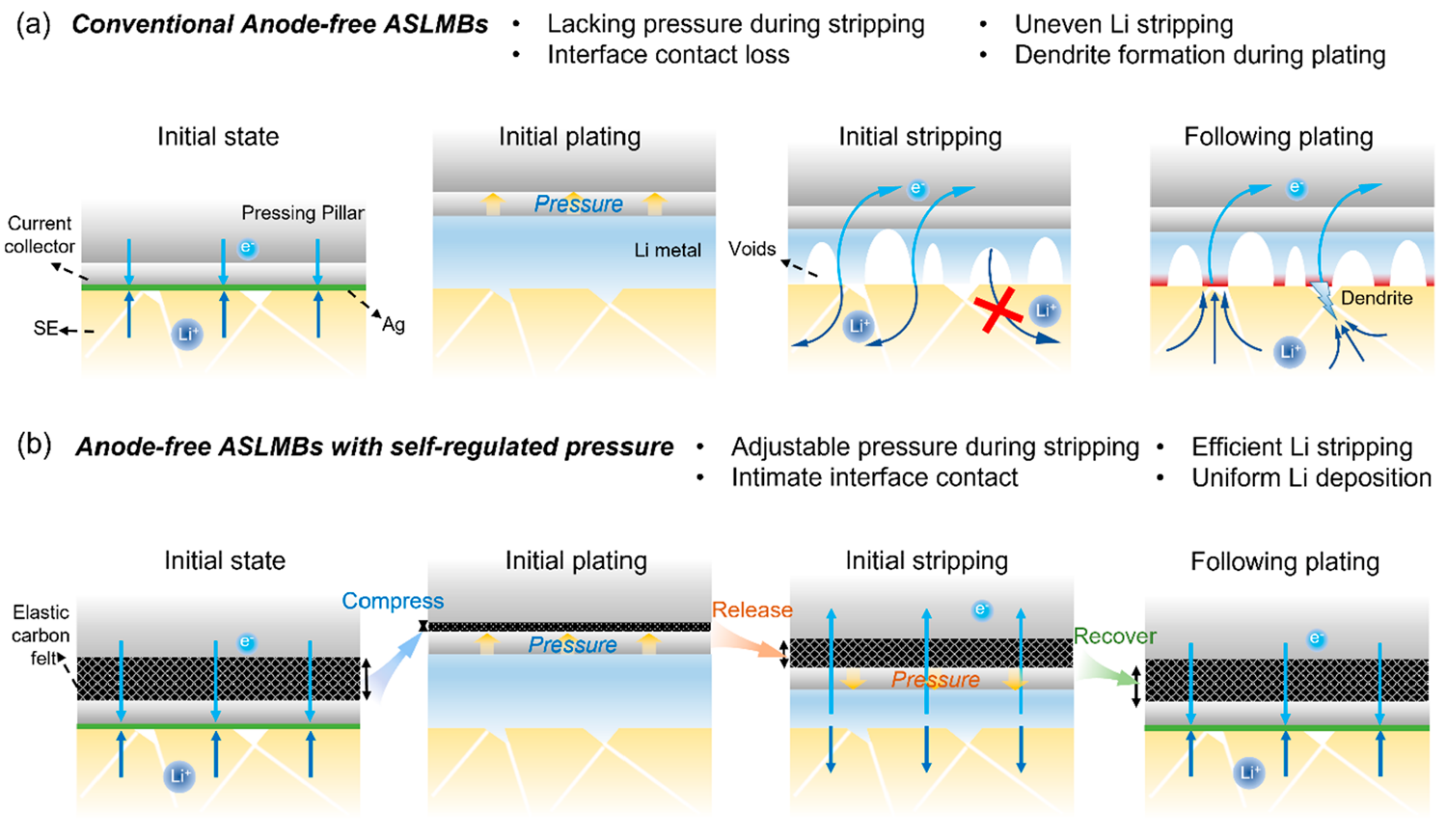
(a) Traditional anode-free ASLMB and (b) anode-free ASLMB with self-regulated pressure during the initial state, initial plating, initial stripping, and subsequent plating.

In contrast, we use an elastic layer, specifically a compressible carbon felt, to self-regulate the pressure within the anode-free ASLMB (Figure 1b). The carbon felt having excellent electron conductivity is positioned between the current collector and the pressing pillar. When the Li metal expands during the plating stage, the carbon felt can be compressed. This compression not only buffers the volume expansion of the Li metal, reducing the risk of the Li metal creeping toward the SE, but also more importantly preserves the pressure generated during plating. As a result, the stored pressure ensures the Li metal remains in close contact with the SE throughout the stripping stage. The capability for timely force adjustment improves the SE–anode interface contact and results in fewer contact loss at the interface. This in turn contributes to a high Li metal stripping efficiency and uniform deposition in subsequent plating processes.^19,20^

We studied the impact of internal pressure control on the stripping efficiency in anode-free ASLMBs with various cathode loadings. Cells were tested under an initial external pressure of 7.5 MPa. To enhance the lithiophilicity, a 20 nm Ag layer was deposited on the SS current collector. Panels a–d of Figure 2 contrast cells with and without carbon felt at cathode loadings of 10 and 20 mg cm^–2^. Without the felt, cells showed fluctuating discharge profiles and rapid capacity fading, with initial ICE of only 58.4% at 10 mg cm^–2^. This indicates challenges in fully stripping the plated Li in the anode-free architecture. In contrast, cells with the carbon felt exhibited stable cycling and dramatically higher ICEs of 83.7% and 81.2% at 10 and 20 mg cm^–2^, respectively. The carbon felt layer markedly improved the Li stripping efficiency.

**Figure 2:**
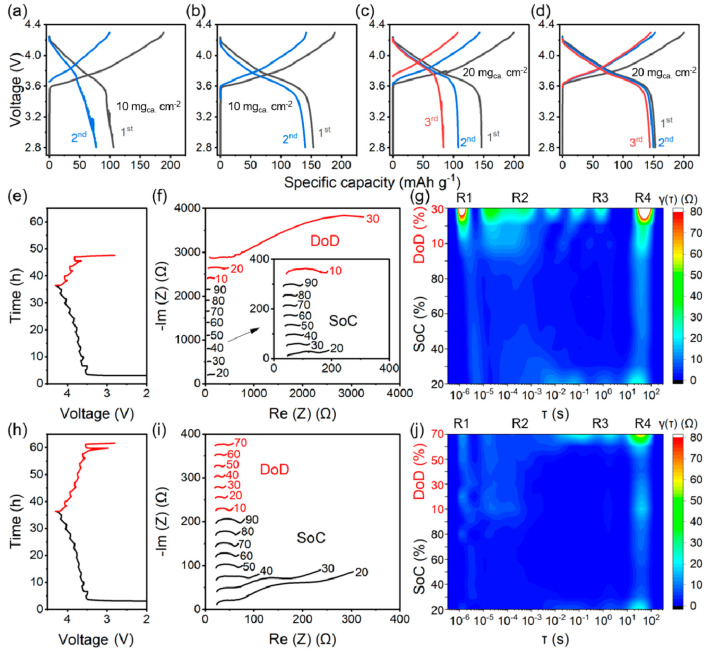
Electrochemical behaviors of anode-free ASLMBs with and without self-regulated pressure. Galvanostatic charge and discharge profiles of anode-free ASLMBs (a) without carbon felt at a cathode mass loading of 10 mg cm^–2^, (b) with carbon felt at a cathode mass loading of 10 mg cm^–2^, (c) without carbon felt at a cathode mass loading of 20 mg cm^–2^, and (d) with carbon felt at a cathode mass loading of 20 mg cm^–2^. Charge–discharge profiles of ASLMBs (e) without and (h) with the carbon felt in the EIS measurement. Nyquist plots of ASLMBS (f) without and (i) with the carbon felt at different SoC and DoD. Two-dimensional color mappings of the DRT curves in ALMBS (g) without and (j) with the carbon felt.

To further investigate the low-stripping efficiency mechanism, we compared EIS at various states of charge and discharge. Cells were cycled at C/20 with a galvanostatic intermittent titration technique (GITT). Without the felt, the impedance drastically increased to >3000 Ω during discharge, explaining the rapid voltage decline. With the felt, the impedance remained below 100 Ω even at a depth of discharge of 70%. The distribution of relaxation time (DRT) analysis showed the increase in impedance in the cell without felt was primarily due to increasing R1, indicating loss of contact at the anode side. In contrast, cells with the felt displayed negligible R1 changes with increases in impedance stemming mainly from cathode diffusion effects. This confirms poor anode contact as the primary issue in ASLMB without the felt, which is effectively mitigated by the compressible interlayer (Figure 2e–j and Figures S2 and S3). In summary, EIS and DRT analyses clearly link poor anode-side contact to impaired stripping in anode-free ASLMBs, which is substantially improved by the self-adjusting pressure enabled through the conductive carbon felt interlayer.

Scanning electron microscopy (SEM) was employed to visualize the interface morphologies on the anode side after the initial stripping. A focused-ion beam was utilized to cut the current collector to expose the cross section of the interface. Figure 3a shows the morphology of the anode side in ASLMB with the carbon felt. There was a dense structure intimately attached to the current collector. The energy dispersive X-ray spectroscopy (EDX) mappings of Fe (Figure 3b) and S (Figure 3c) suggested that this dense structure was the sulfide SE. The intimate contact between the SE and the current collector as well as no excess Li remaining demonstrated the high Li metal stripping efficiency. In comparison, there were porous structures adjacent to the current collector in the ASLMB without the addition of carbon felt (Figure 3d). Huge voids with dimensions of several micrometers were the dominant component. The EDX mappings of Fe (Figure 3e) and S (Figure 3f) proved this porous structure was not the SE. Because there were no other components, the porous structure was assigned to the unstripped Li metal. The voids in the structure were attributed to uneven stripping and the lack of pressure during the stripping process. There was severe contact loss between the Li metal and the current collector, as well. The remaining Li after the stripping proved the low stripping efficiency, and the porous structure of Li metal and the contact loss with the current collector explained the suddenly increased huge resistance. Therefore, with the pressure regulation of the carbon felt, the ASLMB delivered a greatly enhanced Li metal stripping efficiency and intimate contact.

**Figure 3:**
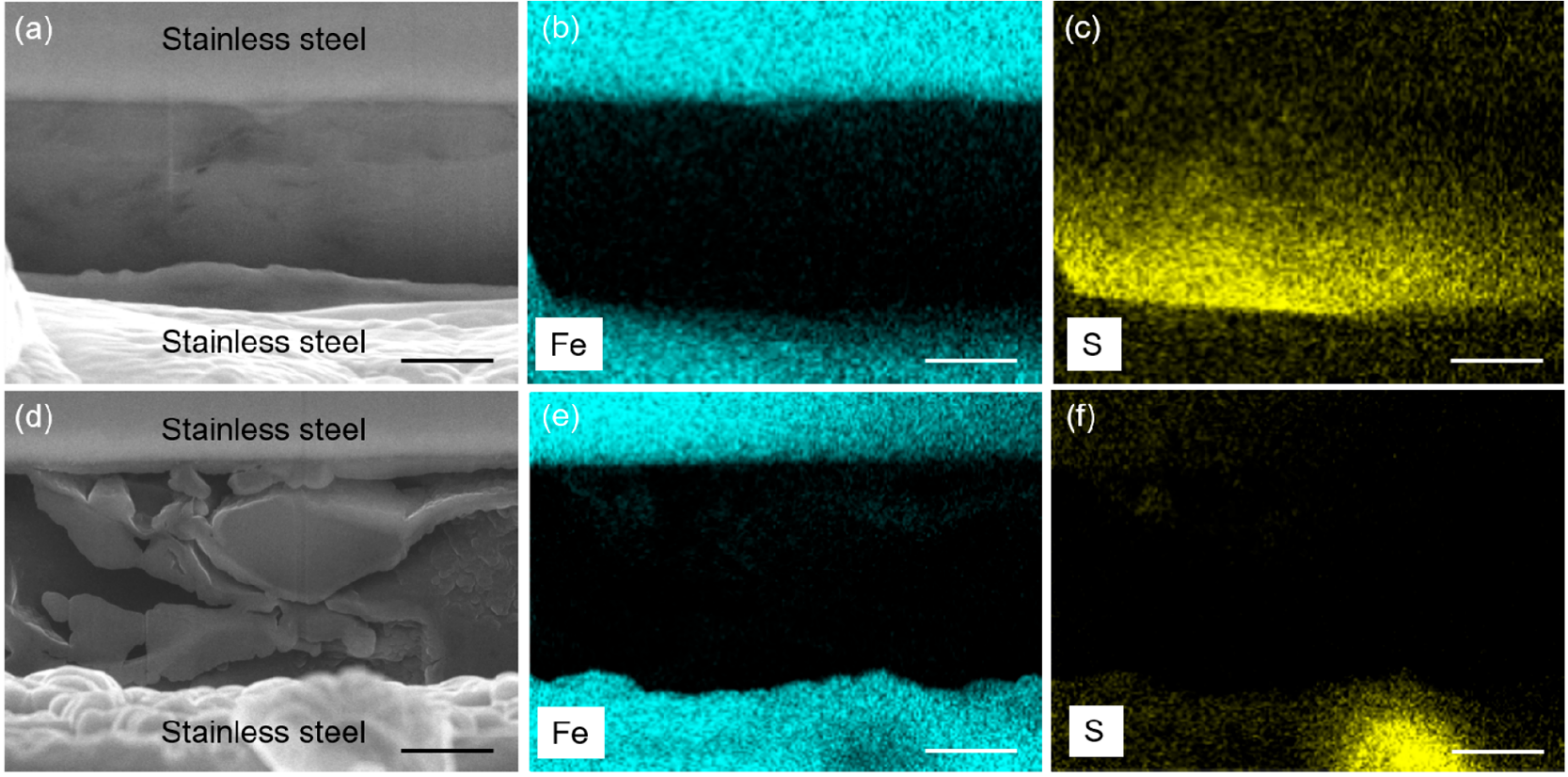
Interface morphologies at the anode side after the initial stripping. (a) SEM image of an anode-free ASLMB with carbon felt and corresponding EDX mappings of (b) Fe and (c) S elements. (d) SEM image of an anode-free ASLMB without carbon felt and corresponding EDX mappings of (e) Fe and (f) S elements. The scale bar is 2 μm.

The CCD of the ASLMB with the addition of carbon felt was evaluated by cycling the ASLMB at stepwise increased current densities from 0.1 to 3 mA cm^–1^ with an increasing rate of 0.1 mA cm^–1^, and the pressure in the cell was operando monitored in the total process (Figure 4a). Overall, the ASLMB did not show a hard short even cycled at 3 mA cm^–2^. However, when the current density exceeded 1.5 mA cm^–2^, there was a typical soft short phenomenon, in which the charge time increased though the current density increased (Figure S4). The soft shortness was attributed to the Li propagation caused by the plating-induced pressure. As the battery was charged and discharged, the inner pressure changed accordingly. The plated Li metal experienced volume expansions when the ASLMB was charged, accompanied by an increase in pressure from 7.65 to 8.05 MPa. During the discharge process, the trend reversed and the pressure decreased to 7.21 MPa. In comparison to the initial value, the reduced pressure after one cycle was mainly caused by the pressure release from the framework (Figure S5). In the following cycles, the pressure regularly fluctuated but gradually decreased, indicating the reaction in the ASLMB was not thorough when it was cycled at a high current density.

**Figure 4:**
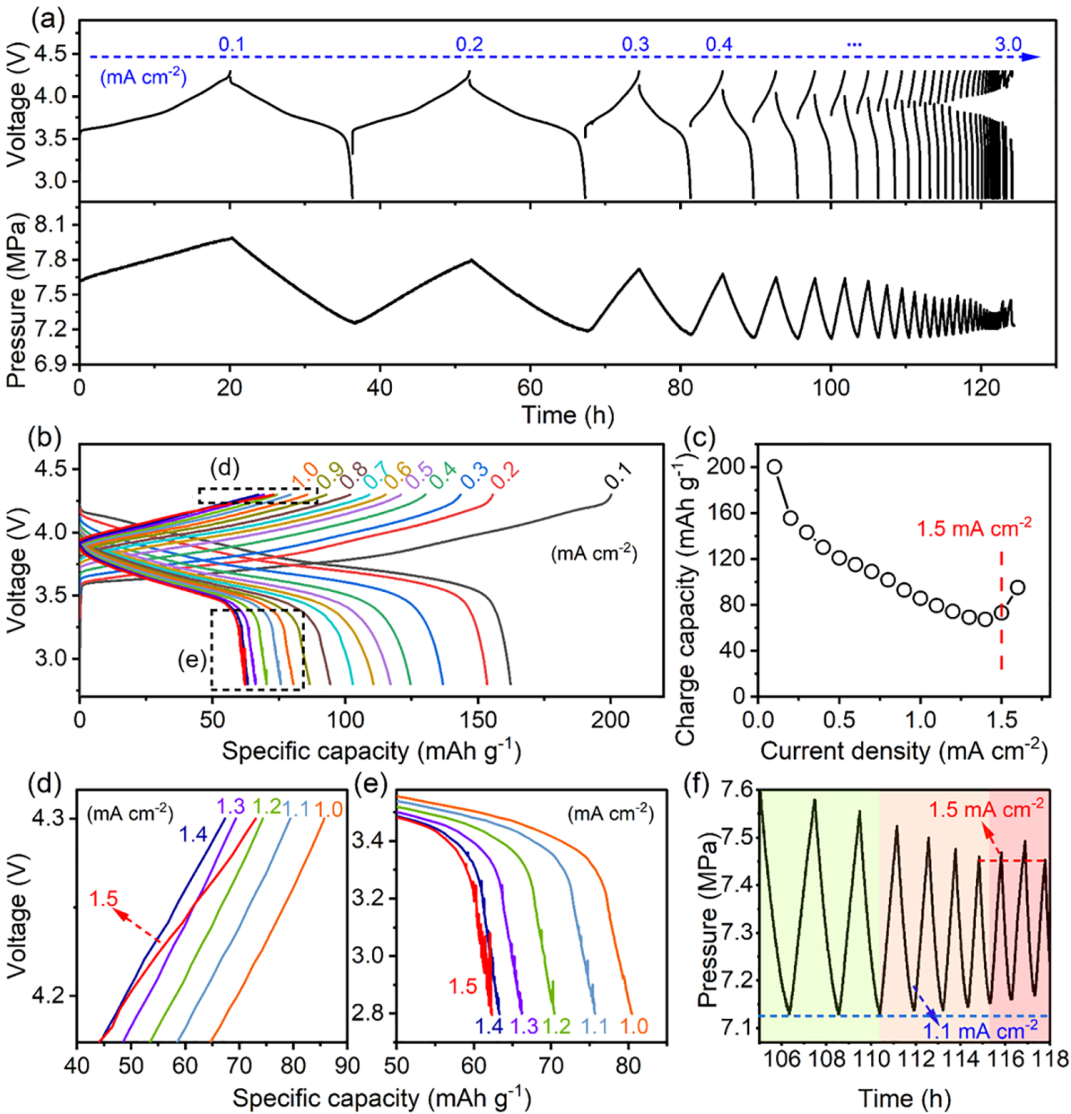
Critical current density evaluation and operando pressure monitoring of an ASLMB with carbon felt. (a) Charge and discharge profiles of the pressure-regulated ASLMB cycled stepwise with the current density increasing from 0.1 to 3.0 mA cm^–2^. The inner pressure evolution was operando recorded. (b) Voltage-specific capacity profiles of the ASLMB cycled at current densities from 0.1 to 1.5 mA cm^–2^. The detailed information is displayed in panels d and e. (c) Specific charge capacities of the ASLMB at different current densities. (d) Discharge and (e) charge profiles of the ASLMB measured at current densities from 1.0 to 1.5 mA cm^–2^. (f) Detailed illustration of the pressure evolution when the ASLMB was cycled at current densities from 0.8 to 1.7 mA cm^–2^.

Figure 4b illustrates the charge–discharge profiles of the ASLMB at a current density of <1.6 mA cm^–2^. As the current rate was increased, the capacity progressively decreased due to the increased overpotential. However, as demonstrated in Figure 4c, the ASLMB that was cycled at 1.5 mA cm^–2^ exhibited a charge capacity increase. The unusual charge profile at 1.5 mA cm^–2^, depicted in Figure 4d, suggested that the ASLMB experienced a mild short circuit. Does this imply that the CCD in this ASLMB is 1.5 mA cm^–2^? Figure 4e compares the discharge profiles. Except for the profile at 1.0 mA cm^–2^, all others displayed voltage fluctuation, a phenomenon akin to that observed in the ASLMB devoid of carbon felt. This implied that the Li metal stripping began to encounter difficulties at 1.1 mA cm^–2^. As previously mentioned, the low stripping efficiency led to a porous Li metal structure that instigated uneven plating in the subsequent cycle, ultimately resulting in a short circuit. Consequently, the CCD, which is predominantly governed by the stripping process, should be identified as 1.0 mA cm^–2^.

Figure 4f displays the detailed pressure evolutions at current densities from 0.8 to 1.7 mA cm^–2^. Before 1.1 mA cm^–2^, the pressure enhancement during charging was gradually reduced due to insufficient reaction and less Li plating caused by the increased overpotential at the higher current density. The pressure at fully discharged states remained stable (blue dashed line), demonstrating good stripping efficiency. However, during cycling at 1.1 mA cm^–2^, the pressure at the full discharge increased a little, demonstrating that Li metal remained. This pressure kept increasing at higher current densities. When measured at 1.5 mA cm^–2^, the pressure enhancement increased slightly (red dashed line) at charge, contrary to the previous trend. This pressure enhancement was attributed to the increased amount of Li metal due to the prolonged charging time. Our previous work has proven there were still Faradaic reactions that occurred in the soft-shorted ASLMBs.^21^ Therefore, the pressure evolution further confirmed that the CCD was dominated by insufficient stripping, which is in advance of the plating-induced short circuit.

The long-term cycling performance was investigated. Figure 5a displays the cycling performance of the anode-free ASLMBs with the carbon felt at a current rate of C/5 after activation at C/20 for one cycle. The ASLMB delivered high discharge capacities of ∼130 mAh g^–1^ and an average CE of >99.5% in the initial 30 cycles, attributed to the self-adjusted pressure enabled by the carbon felt. However, a capacity decay was observed in the subsequent 70 cycles, decreasing to 72 mAh g^–1^, which signifies the diminishing effectiveness of carbon felt over time. Figure 5b shows the charge–discharge profiles during the test. Stable cycling was performed in the initial 30 cycles. After that, although the charging time gradually decreased, the charging process showed normal, and no soft short was observed. In contrast, the discharge profiles exhibited sudden voltage decreases accompanied by greatly reduced discharge capacities. Note that the overpotential during discharge showed no obvious change, suggesting the gradually reduced charging time was caused by the low-lithiation states of the NMC during the last discharge process. Therefore, the failure of this ASLMB was strongly related to the stripping process. Considering the good cycling performance in the initial 30 cycles, the carbon felt played a significant role in this behavior difference. In Figure S6, which displays the cycling stability and capacity of the control sample without carbon, the capacity significantly decreases to ∼50 mAh/g after the second cycle. This decline is attributed to interface reactions and a reduced stripping efficiency.

**Figure 5:**
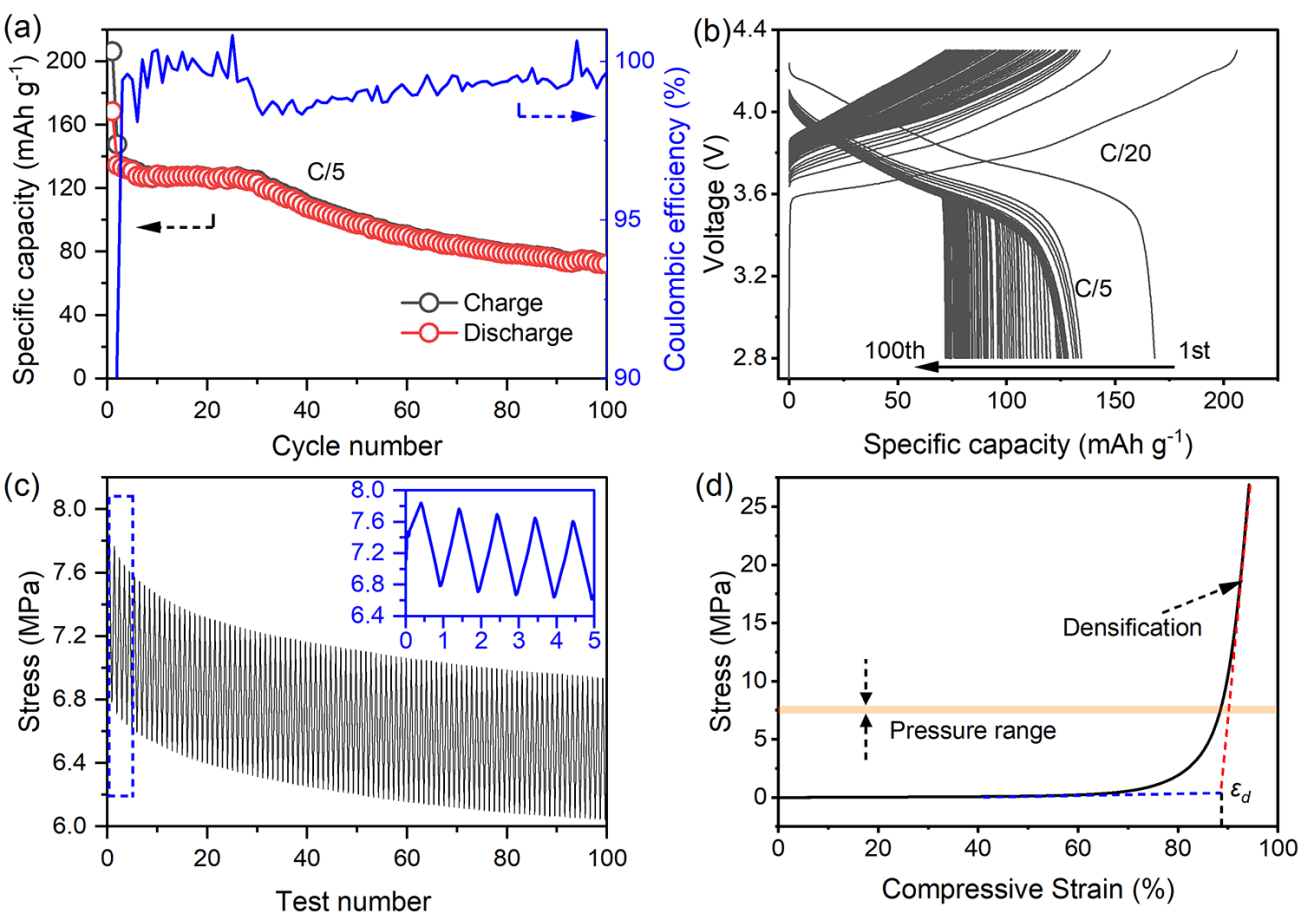
Cycling performance evaluation and failure mechanism analysis. (a) Cycling performance and (b) corresponding charge–discharge profiles of the ASLMB with the carbon felt at a current rate of C/5. (c) Fatigue test of the carbon felt. The inset shows the details in the initial five cycles. (d) Compressive stress–strain profile of the carbon felt.

The carbon felt underwent continued compress–release processes in the ASLMBs. Thus, their compressive performances were evaluated. Figure 5c depicts the compression fatigue behavior of the carbon felt. After application of an initial pressure of 7.5 MPa, the pressure head moved axially down and up at a rate of 1 μm/s within a displacement of 15 μm for 100 cycles, and the pressure was monitored. Fifteen micrometers was the theoretical thickness of the deposited Li metal equal to the areal capacity of 3 mAh cm^–2^. Overall, the mean stress gradually decayed from 7.5 to 6.5 MPa after 100 cycles. The stress profile in the inset shows the carbon felt underwent linear deformation upon repeated compression and release. The decreased mean stress demonstrated there is energy loss in the carbon felt during the releasing process, i.e., the stripping process. Figure 5d displays the compression stress–strain profile of the carbon felt inside the cell for ASLMBs. After a long plateau up to ∼65% of strain, the strain delivered a sharp increase, which was attributed to the collapsing and further densification of the carbon felt. The densification strain, ε_d_, was ∼89%, at which point the carbon felt was completely crushed. The pressure range in the ASLMBs was highlighted, and the corresponding strain was close to the ε_d_. Therefore, the carbon felt started to crush after the initial pressing and kept aging in the repeated compression and release, which explained the failure of the ASLMB after 30 cycles in the long-term cycling test. Though failing in long-term cycling, the carbon felt layer successfully demonstrated the strategy we proposed in enhancing the stripping efficiency of the anode-free ASLMBs. Table S1 compares the reported full cell performance of the anode-free ASLMBs using sulfide SEs. The carbon felt-assisted anode-free ASLMBs delivered an excellent room-temperature full cell performance. We believe the long cycling performance can be achieved through carbon felt mechanical fatigue modification.

In summary, our in-depth analysis of the failure mechanism of ASLMBs has underscored the crucial role of interface contact between the anode and SE in the stripping efficiency. Our findings suggested a strategy for enhancing this efficiency by automatically regulating the internal pressure during plating and stripping using an elastic electrically conductive interlayer like a compressible carbon felt. During plating, the felt is compressed to accommodate lithium expansion. During stripping, it releases pressure to ensure consistent lithium–solid electrolyte contact, preventing interfacial voids and/or gaps. Impedance spectroscopy and DRT analysis confirmed the felt mitigates contact loss and an increase in impedance at the anode.

The morphological characteristics of the anode side after complete stripping showed that the carbon felt greatly improved the Li stripping efficiency, unlike the control group that exhibited unstripped Li metal with a porous structure. As a result, the initial Coulombic efficiency increased significantly from 58.4% to 83.7%, and the average CE was maintained above 99% in subsequent cycles. With the inclusion of the carbon felt, the anode-free ASLMBs achieved a high critical current density (CCD) of 1.0 mA cm^–2^, and pressure monitoring evidence indicated that the CCD was strongly tied to the stripping process. Further evaluation of the compression behavior of the carbon felt provided insights into its long-term behavior within the anode-free ASLMBs. This behavior could potentially be further improved by introducing an elastic layer with a higher compression durability. The proposed method has promising implications for large-scale anode-free ASLMB applications, such as pouch cells, where it could automatically regulate pressure, thus enhancing both the performance and the longevity of these high-energy density and safer batteries.

## Supporting Information Available

Supplementary electrochemical results, including impedance analyses, charge and discharge profiles, and pressure monitoring data for cells with and without the carbon felt interlayer, and a table compiling performance metrics from previous anode-free sulfide solid electrolyte batteries that allows comparison with the results achieved here (PDF)

## References

[ref1] International Energy Agency. Global EV Outlook 2020. 2020. 10.1787/d394399e-en

[ref2] Janek J.; Zeier W. G. Challenges in speeding up solid-state battery development. Nature Energy 2023, 8 (3), 230–240. 10.1038/s41560-023-01208-9.

[ref3] Sun Y.-K. Promising All-Solid-State Batteries for Future Electric Vehicles. ACS Energy Letters 2020, 5 (10), 3221–3223. 10.1021/acsenergylett.0c01977.

[ref4] Lee Y.-G.; Fujiki S.; Jung C.; Suzuki N.; Yashiro N.; Omoda R.; Ko D.-S.; Shiratsuchi T.; Sugimoto T.; Ryu S.; et al. High-energy long-cycling all-solid-state lithium metal batteries enabled by silver–carbon composite anodes. Nature Energy 2020, 5 (4), 299–308. 10.1038/s41560-020-0575-z.

[ref5] Huang W.-Z.; Zhao C.-Z.; Wu P.; Yuan H.; Feng W.-E.; Liu Z.-Y.; Lu Y.; Sun S.; Fu Z.-H.; Hu J.-K.; et al. Anode-Free Solid-State Lithium Batteries: A Review. Adv. Energy Mater. 2022, 12 (26), 2201044 10.1002/aenm.202201044.

[ref6] Tong Z.; Bazri B.; Hu S.-F.; Liu R.-S. Interfacial chemistry in anode-free batteries: challenges and strategies. Journal of Materials Chemistry A 2021, 9 (12), 7396–7406. 10.1039/D1TA00419K.

[ref7] Ning Z.; Jolly D. S.; Li G.; De Meyere R.; Pu S. D.; Chen Y.; Kasemchainan J.; Ihli J.; Gong C.; Liu B.; et al. Visualizing plating-induced cracking in lithium-anode solid-electrolyte cells. Nat. Mater. 2021, 20 (8), 1121–1129. 10.1038/s41563-021-00967-8. 33888903

[ref8] Lewis J. A.; Cortes F. J. Q.; Liu Y.; Miers J. C.; Verma A.; Vishnugopi B. S.; Tippens J.; Prakash D.; Marchese T. S.; Han S. Y.; et al. Linking void and interphase evolution to electrochemistry in solid-state batteries using operando X-ray tomography. Nat. Mater. 2021, 20 (4), 503–510. 10.1038/s41563-020-00903-2. 33510445

[ref9] Xu R.; Han F.; Ji X.; Fan X.; Tu J.; Wang C. Interface engineering of sulfide electrolytes for all-solid-state lithium batteries. Nano Energy 2018, 53, 958–966. 10.1016/j.nanoen.2018.09.061.

[ref10] Han F.; Westover A. S.; Yue J.; Fan X.; Wang F.; Chi M.; Leonard D. N.; Dudney N. J.; Wang H.; Wang C. High electronic conductivity as the origin of lithium dendrite formation within solid electrolytes. Nature Energy 2019, 4 (3), 187–196. 10.1038/s41560-018-0312-z.

[ref11] Albertus P.; Anandan V.; Ban C.; Balsara N.; Belharouak I.; Buettner-Garrett J.; Chen Z.; Daniel C.; Doeff M.; Dudney N. J.; et al. Challenges for and Pathways toward Li-Metal-Based All-Solid-State Batteries. ACS Energy Letters 2021, 6 (4), 1399–1404. 10.1021/acsenergylett.1c00445.

[ref12] Kazyak E.; Wang M. J.; Lee K.; Yadavalli S.; Sanchez A. J.; Thouless M. D.; Sakamoto J.; Dasgupta N. P. Understanding the electro-chemo-mechanics of Li plating in anode-free solid-state batteries with operando 3D microscopy. Matter 2022, 5 (11), 3912–3934. 10.1016/j.matt.2022.07.020.

[ref13] Cao D.; Sun X.; Li Y.; Anderson A.; Lu W.; Zhu H. Long-Cycling Sulfide-Based All-Solid-State Batteries Enabled by Electrochemo-Mechanically Stable Electrodes. Adv. Mater. 2022, 34 (24), 2200401 10.1002/adma.202200401. 35405025

[ref14] Lee C.; Han S. Y.; Lewis J. A.; Shetty P. P.; Yeh D.; Liu Y.; Klein E.; Lee H.-W.; McDowell M. T. Stack Pressure Measurements to Probe the Evolution of the Lithium–Solid-State Electrolyte Interface. ACS Energy Letters 2021, 6 (9), 3261–3269. 10.1021/acsenergylett.1c01395.

[ref15] Doux J.-M.; Nguyen H.; Tan D. H. S.; Banerjee A.; Wang X.; Wu E. A.; Jo C.; Yang H.; Meng Y. S. Stack Pressure Considerations for Room-Temperature All-Solid-State Lithium Metal Batteries. Adv. Energy Mater. 2020, 10 (1), 1903253 10.1002/aenm.201903253.

[ref16] Chen Y.; Wang Z.; Li X.; Yao X.; Wang C.; Li Y.; Xue W.; Yu D.; Kim S. Y.; Yang F.; et al. Li metal deposition and stripping in a solid-state battery via Coble creep. Nature 2020, 578 (7794), 251–255. 10.1038/s41586-020-1972-y. 32015545

[ref17] Feng M.; Yang C.-T.; Qi Y. The Critical Stack Pressure to Alter Void Generation at Li/Solid-Electrolyte Interfaces during Stripping. J. Electrochem. Soc. 2022, 169 (9), 090526 10.1149/1945-7111/ac91aa.

[ref18] Cao D.; Ji T.; Singh A.; Bak S.; Du Y.; Xiao X.; Xu H.; Zhu J.; Zhu H. Unveiling the Mechanical and Electrochemical Evolution of Nanosilicon Composite Anodes in Sulfide-Based All-Solid-State. Batteries. 2023, 13 (14), 2203969 10.1002/aenm.202203969.

[ref19] Wan T. H.; Saccoccio M.; Chen C.; Ciucci F. Influence of the Discretization Methods on the Distribution of Relaxation Times Deconvolution: Implementing Radial Basis Functions with DRTtools. Electrochim. Acta 2015, 184, 483–499. 10.1016/j.electacta.2015.09.097.

[ref20] Li X.; Liang J.; Kim J. T.; Fu J.; Duan H.; Chen N.; Li R.; Zhao S.; Wang J.; Huang H.; et al. Highly Stable Halide-Electrolyte-Based All-Solid-State Li–Se Batteries. Adv. Mater. 2022, 34 (20), 2200856 10.1002/adma.202200856. 35365923

[ref21] Cao D.; Zhang K.; Li W.; Zhang Y.; Ji T.; Zhao X.; Cakmak E.; Zhu J.; Cao Y.; Zhu H. Nondestructively Visualizing and Understanding the Mechano-Electro-Chemical Origins of "Soft Short" and "Creeping" in All-Solid-State Batteries. Adv. Funct. Mater. 2023, 2307998 10.1002/adfm.202307998.

