# Supplementary material for: Enhancing Lithium Stripping Efficiency in Anode-Free Solid-State Batteries through Self-Regulated Internal Pressure
Source: Nano Lett. 2023 Oct 11;23(20):9392–8. doi: 10.1021/acs.nanolett.3c02713 (PMC10621033; doi:10.1021/acs.nanolett.3c02713)
Supplement: Supplementary file 1 — nl3c02713_si_001.pdf [file nl3c02713_si_001.pdf]

Supporting Information

Enhancing Lithium Stripping Efficiency in Anode-Free Solid-State Batteries through Self-Regulated Internal Pressure

Daxian Cao[1], Tongtai Ji[1], Zhengxuan Wei[1], Wentao Liang[1], Ruobing Bai[1], Kenneth S. Burch[2], Michael Geiwitz[2], Hongli Zhu[1,*]

[1]Department of Mechanical and Industrial Engineering, Northeastern University, 360 Huntington Avenue, Boston, Massachusetts 02115, United States

[2]Department of Physics, Boston College, Chestnut Hill, Massachusetts 02467, USA

Experiment section

Material Preparation:

Argyrodite type sulfide solid electrolyte, $Li_{5.4}PS_{4.4}Cl_{1.6}$, was used in this study. Li_2S (Sigma–Aldrich, 99.98%), P_2S_5 (Sigma– Aldrich, 99%), and LiCl (Sigma–Aldrich, 99%) were used as received. The synthesis process has been reported in our previous work.[1] The solid electrolyte was grind into powder before using. The cathode active material, single-crystal NMC 811 (Nanoramic Inc, USA), was coated by Li_2SiO_x through a wet chemical method.[2] The composite cathode material was prepared by mixing 75 wt% Li_2SiO_x-coated single-crystal NMC 811 with 25 wt% $Li_{5.4}PS_{4.4}Cl_{1.6}$ solid electrolyte through manually grinding for 20 mins in the mortar. The Ag deposition on stainless steel current collector was conducted in the e-beam deposition equipment (Angstrom Engineering, Inc.) inside a unique cleanroom-in-a-glovebox.[3] The chamber was pumped to a base pressure of 2×10^{-6} Torr, and then 20 nm of silver was deposited at a rate of 5

Å/s. Carbon felt (GFD 4.65 EA, Sigracell) was cut into circles with a diameter of 12.7 mm and prepared for use.

Material Characterizations

The cross-sectional morphology was collected on a high-resolution SEM/FIB-FEI Scios DualBeam system. TEM and EDS mapping images were collected on the Cs-corrected TEM/STEM-FEI TitanThemis 300. The compression test of the carbon felt was conducted on the Universal testing machine (34TM-50, Instron) with a compression speed of 1 μm/S. The compression fatigue test was conducted on the same device. A 7.5 MPa of pressure was first placed on the carbon felt. The pressure was monitored during moving the pressure head down and up under the rate of 1 μm/S within a displacement of 15 μm for one hundred cycles.

All Solid State Battery Assembling

The all solid state battery was assembled by the cold pressing method. For the anode-free full cells, firstly, 127 mg solid electrolyte was put into a polyether ether ketone (PEEK) die with a diameter of 12.7 mm. The solid electrolyte was prepressed under the pressure of 10 MPa by two stainless steel pillars. 12.7 mg (cathode mass loading of ~10 mg/cm^2) or 25.4 mg (cathode mass loading of ~20 mg/cm^2) of composite cathode material was uniformly cast on one side of the solid electrolyte and further covered by an aluminum foil as the current collector. The cell was pressed under the pressure of 450 MPa. Then, the pristine stainless steel current collector or Ag coated stainless steel current collector was placed on the other side of the solid electrolyte. For the cell with carbon felt, the carbon felt was put between the stainless steel current collector and stainless steel pillar. Three kinds of symmetric cells (Cathode|SE|Cathode , SS|SE|SS, and Li|SE|Li) were fabricated with a similar process. 25.4 mg of composite NMC cathode material, Ag coated stainless steel current collector, or Li foil (thickness ~150 μm) were put on both side of the solid electrolyte (127 mg) to

fabricate the symmetric cells. A 450 MPa fabrication pressure was used in this process. Note, for the Li|SE|Li symmetric cell, Li foils were added after compressing the cell with the pressure of 450 MPa. A 7.5 MPa of stacking pressure was applied during the test by an additional framework.

Electrochemical characterization

EIS and *in-operando* EIS measurement: EIS was conducted on the Biologic SP150 potentiostat (Biologic) with an AC amplitude of 10 mV from 1 MHz to 10 mHz. The *in-operando* EIS was measured by the same device. The cell was galvanostatically cycled under the rate of C/20. EIS was tested every one hour of charge or discharge after one hour of rest.

Rate and Cycling Performance and *in-operando* Pressure monitor: The rate performance for the full cell was tested by the LANDT 8-channel tester (Wuhan LAND Electronic Co., Ltd.). The cell was galvanostatically cycled between 2.8-4.3 V with the current density from 0.1 mA/cm^2 to 3.0 mA/cm^2 with an increase of 0.1 mA/cm^2 after each cycle. A pressure sensor (DYMH-105, Daysensor) was placed between the stainless steel pillar of solid state battery and the framework to monitor and record the pressure change during the rate test of the battery. For the long cycling test, the cell was first activated under the rate of C/20 for one cycle and then cycled under C/5.

Supporting figures

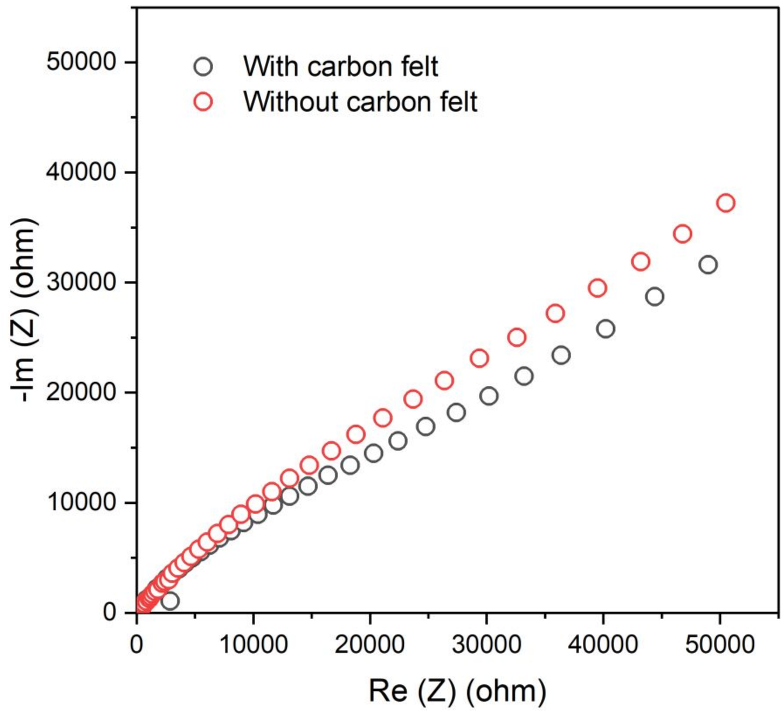

Figure S1. Nyquist plots of the anode-free ASLMBs with and without the carbon felt before cycling.

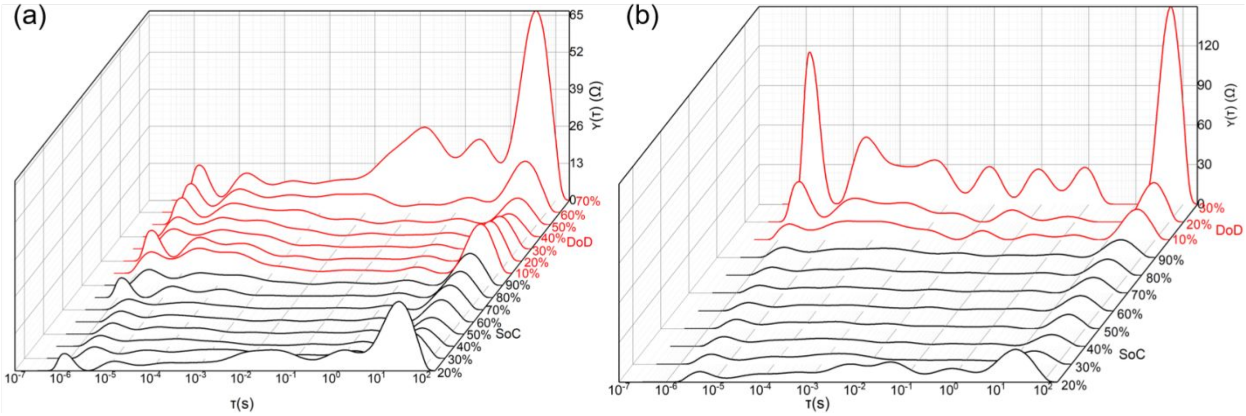

Figure S2. DRT results of the anode-free ASLMBs (a) with and (b) without the carbon felt at different SoC and DoD.

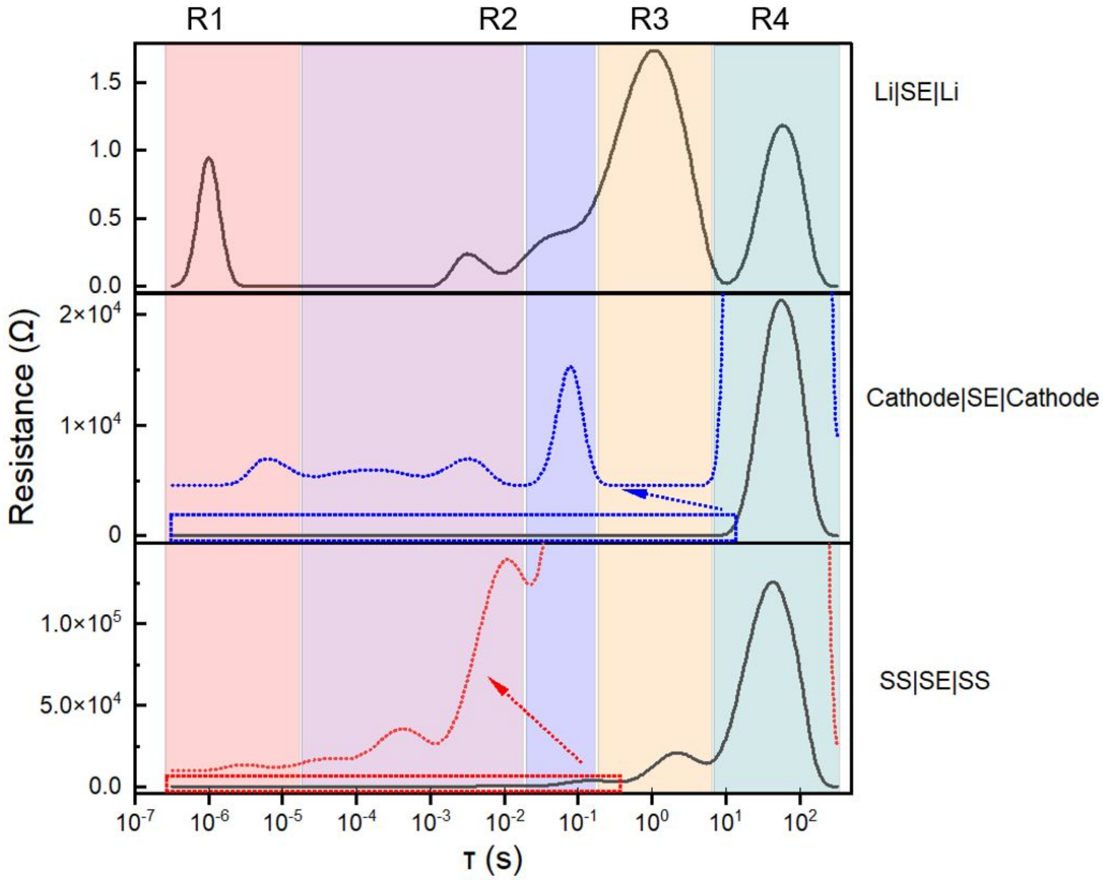

Figure S3. DRT results of symmetric cells of Li|SE|Li, Cathode|SE|Cathode, and SS|SE|SS.

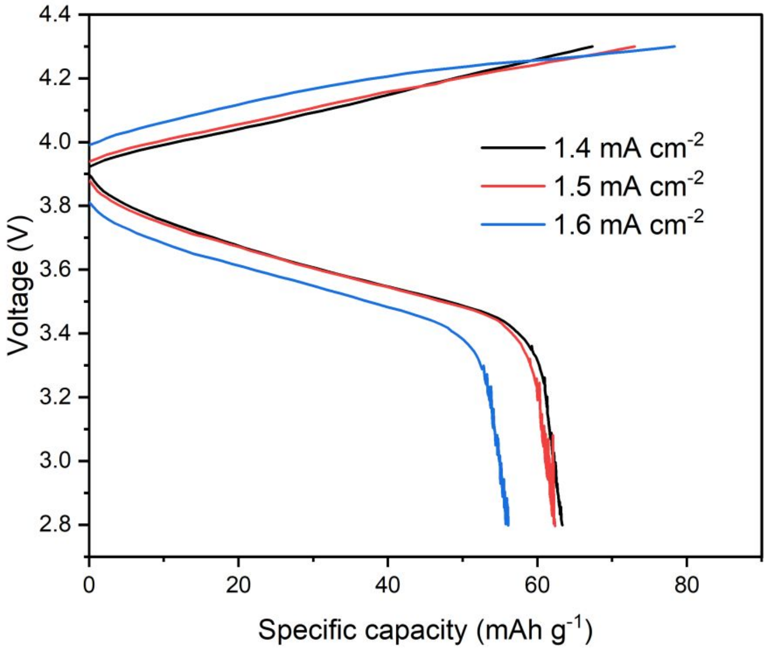

Figure S4. Charge-discharge profiles of the anode-free ASLMB cycled at 1.4, 1.5, and 1.6 mA cm^{-2}, showing the soft short phenomena.

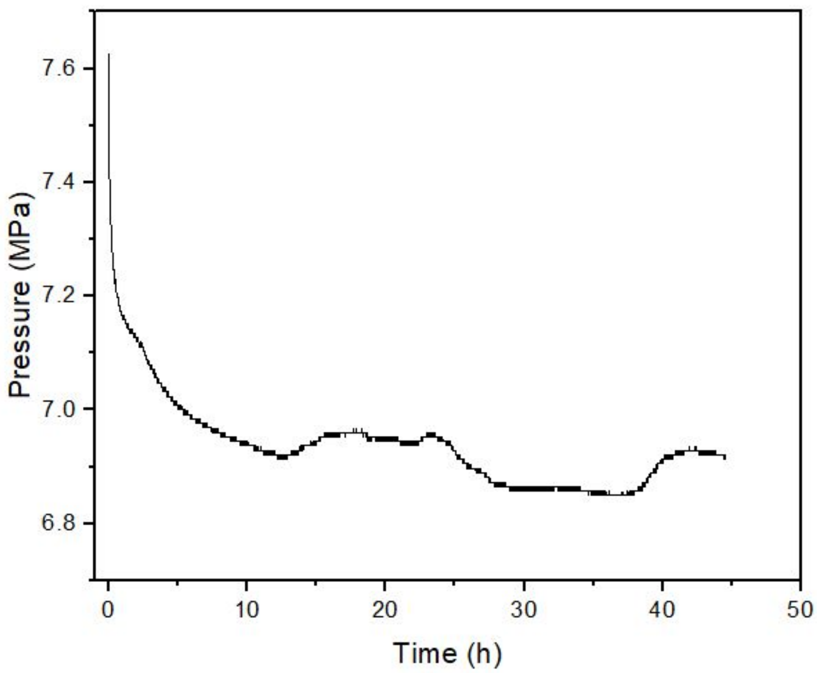

Figure S5. Pressure monitoring of the framework during rest to show the pressure release.

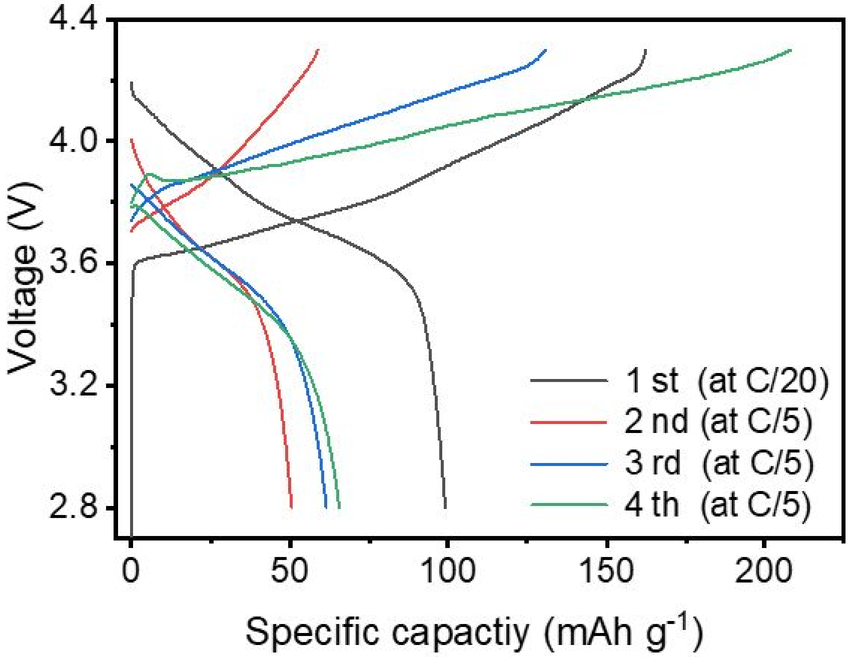

Figure S6. Charge-discharge profiles of the ASLMB without the carbon felt

Table S1. Full cell battery performance comparison with reported anode-free ASLMBs based on sulfide SEs.

No.	Anode@Current collector	Areal capacity (mAh cm^{-2})	Initial coulombic efficiency (%)	Average coulombic efficiency (%)	Capacity retention	Test temperature	Ref.
1	Weakly etched stainless steel	2.92	~95.6	93.4	73.6% after 5 cycles	60 °C	[4]
	Strongly etched stainless steel	2.92	~90.0	85.6	35.4% after 5 cycles	60 °C	
2	Carbon paper	4.0	~55.0	/	/	RT	[5]
3	Te@Cu	2.4	83.0	99	80% after 50 cycles	RT	[6]
4	Ag-C@Stainless steel	6.8	~91.5	99.8	95% after 600 cycles, 89% after 1000 cycles	60 °C	[7]
5	Graphite-SE@Cu	0.75	~68.6	98.8	38% after 80 cycles	RT	[8]
		1	~71.8	98.5	36% after 70 cycles	RT	
		1.25	~71.1	97.5	27% after 55 cycles	RT	
**6**	**Ag@Stainless steel**	**3**	**58.3**	**/**	**53% after 3 cycles**	**RT**	**This work**
	**Ag@Stainless steel-carbon felt**	**3**	**83.7**	**99.5**	**92.3% after 30 cycles, 55.4% after 100 cycles**	**RT**	**This work**

Reference

(1) Sun, X.; Cao, D.; Wang, Y.; Ji, T.; Liang, W.; Zhu, H. All-Solid-State Li–S Batteries Enhanced by Interface Stabilization and Reaction Kinetics Promotion through 2D Transition Metal Sulfides. *Advanced Materials Interfaces* **2022**, *9* (20), 2200539. DOI: 10.1002/admi.202200539.
(2) Cao, D.; Ji, T.; Singh, A.; Bak, S.; Du, Y.; Xiao, X.; Xu, H.; Zhu, J.; Zhu, H. Unveiling the Mechanical and Electrochemical Evolution of Nanosilicon Composite Anodes in Sulfide-Based All-Solid-State Batteries. *Advanced Energy Materials* **2023**, *13* (14), 2203969. DOI: 10.1002/aenm.202203969.
(3) Gray, M. J.; Kumar, N.; O'Connor, R.; Hoek, M.; Sheridan, E.; Doyle, M. C.; Romanelli, M. L.; Osterhoudt, G. B.; Wang, Y.; Plisson, V.; et al. A cleanroom in a glovebox. *Review of Scientific Instruments* **2020**, *91* (7). DOI: 10.1063/5.0006462.
(4) Gu, D.; Kim, H.; Lee, J.-H.; Park, S. Surface-roughened current collectors for anode-free all-solid-state batteries. *Journal of Energy Chemistry* **2022**, *70*, 248-257. DOI: 10.1016/j.jechem.2022.02.034.
(5) Huang, W.-Z.; Liu, Z.-Y.; Xu, P.; Kong, W.-J.; Huang, X.-Y.; Shi, P.; Wu, P.; Zhao, C.-Z.; Yuan, H.; Huang, J.-Q.; et al. High-areal-capacity anode-free all-solid-state lithium batteries enabled by interconnected carbon-reinforced ionic-electronic composites. *Journal of Materials Chemistry A* **2023**, *11* (24), 12713-12718, 10.1039/D3TA00121K. DOI: 10.1039/D3TA00121K.
(6) Wang, Y.; Liu, Y.; Nguyen, M.; Cho, J.; Katyal, N.; Vishnugopi, B. S.; Hao, H.; Fang, R.; Wu, N.; Liu, P.; et al. Stable Anode-Free All-Solid-State Lithium Battery through Tuned Metal Wetting on the Copper Current Collector. *Advanced Materials* **2023**, *35* (8), 2206762. DOI: 10.1002/adma.202206762.
(7) Lee, Y.-G.; Fujiki, S.; Jung, C.; Suzuki, N.; Yashiro, N.; Omoda, R.; Ko, D.-S.; Shiratsuchi, T.; Sugimoto, T.; Ryu, S.; et al. High-energy long-cycling all-solid-state lithium metal batteries enabled by silver–carbon composite anodes. *Nature Energy* **2020**, *5* (4), 299-308. DOI: 10.1038/s41560-020-0575-z.
(8) Xing, X.; Li, Y.; Wang, S.; Liu, H.; Wu, Z.; Yu, S.; Holoubek, J.; Zhou, H.; Liu, P. Graphite-Based Lithium-Free 3D Hybrid Anodes for High Energy Density All-Solid-State Batteries. *ACS Energy Letters* **2021**, *6* (5), 1831-1838. DOI: 10.1021/acsenergylett.1c00627.
